# Bidirectional TRP/L Type Ca^2+^ Channel/RyR/BK_Ca_ Molecular and Functional Signaloplex in Vascular Smooth Muscles

**DOI:** 10.3390/biom13050759

**Published:** 2023-04-27

**Authors:** Dariia O. Dryn, Mariia I. Melnyk, Donal Melanaphy, Igor V. Kizub, Christopher D. Johnson, Alexander V. Zholos

**Affiliations:** 1 O.O. Bogomoletz Institute of Physiology, National Academy of Sciences of Ukraine, 01024 Kyiv, Ukraine; gribovamari@gmail.com; 2 ESC “Institute of Biology and Medicine”, Taras Shevchenko National University of Kyiv, 01601 Kyiv, Ukraine; avzholos@knu.ua; 3 Centre for Experimental Medicine, School of Medicine, Dentistry and Biomedical Sciences, Queen’s University Belfast, Belfast BT9 7BL, UK; 4 Division of Endocrinology and Metabolism, Department of Medicine, University of California San Diego, La Jolla, CA 92093, USA; igor.kizub@gmail.com; 5 Centre for Biomedical Sciences Education, Queen’s University Belfast, Whitla Medical Building, Belfast BT9 7BL, UK; c.johnson@qub.ac.uk

**Keywords:** vascular smooth muscles, TRPV4 channels, TRPM8 channels, BK_Ca_ channels, L-type Ca^2+^-channels, vasodilatation, vasoconstriction

## Abstract

TRP channels are expressed both in vascular myocytes and endothelial cells, but knowledge of their operational mechanisms in vascular tissue is particularly limited. Here, we show for the first time the biphasic contractile reaction with relaxation followed by a contraction in response to TRPV4 agonist, GSK1016790A, in a rat pulmonary artery preconstricted with phenylephrine. Similar responses were observed both with and without endothelium, and these were abolished by the TRPV4 selective blocker, HC067047, confirming the specific role of TRPV4 in vascular myocytes. Using selective blockers of BK_Ca_ and L-type voltage-gated Ca^2+^ channels (Ca_L_), we found that the relaxation phase was inducted by BK_Ca_ activation generating STOCs, while subsequent slowly developing TRPV4-mediated depolarisation activated Ca_L_, producing the second contraction phase. These results are compared to TRPM8 activation using menthol in rat tail artery. Activation of both types of TRP channels produces highly similar changes in membrane potential, namely slow depolarisation with concurrent brief hyperpolarisations due to STOCs. We thus propose a general concept of bidirectional TRP-Ca_L_-RyR-BK_Ca_ molecular and functional signaloplex in vascular smooth muscles. Accordingly, both TRPV4 and TRPM8 channels enhance local Ca^2+^ signals producing STOCs via TRP–RyR–BK_Ca_ coupling while simultaneously globally engaging BK_Ca_ and Ca_L_ channels by altering membrane potential.

## 1. Introduction

Vascular tone is tightly controlled by a plethora of ion channels, pumps, and exchangers in the plasma membrane and the sarcoplasmic reticulum (SR) of vascular smooth muscle cells (SMCs). Ion channels function as main gates for calcium entry and release and, of particular relevance, contribute to the regulation of intracellular Ca^2+^ concentration ([Ca^2+^]_i_), one of the main factors of SMC membrane potential regulation and contractile state. Among the various calcium entry pathways present in SMCs, pivotal roles are played by L-type voltage-gated Ca^2+^ channels (Ca_L_) and non-selective cation channels (NSCCs) [1,2]. Most notably, NSCC includes multiple types of polymodal transient receptor potential (TRP) cation channels. These channels are involved in the mechanisms of both vasodilation and vasoconstriction, and hence in various pathological states, such as hypertension, an occlusive vascular disease associated with atherosclerosis, vascular injury, pulmonary arterial hypertension, inflammation, and diabetes [3,4]. Essential contributions of specific types of ion channels, TRP channels, K^+^ channels, Ca_L_, and SR Ca^2+^ release channels in the regulation of myogenic tone have been demonstrated in numerous studies, but their interaction is less well understood [5,6].

Mammalian TRP cation channels form a superfamily that consists of 28 members that are heterogeneously expressed in different cell types [7]. This class of Ca^2+^-permeable channels participate in numerous very important cell functions, notably in sensory perception, regulation of Ca^2+^ and Mg^2+^ homoeostasis, signal transduction and cell growth, and death and cycle regulation. In blood vessels, these proteins are involved in the regulation of vascular tone, particularly in smooth muscle cells, where they regulate cell proliferation and contractility. In addition, endothelial TRP channels regulate vascular wall permeability and angiogenesis. As cellular polymodal sensors, they are stimulated by a variety of factors, including physical factors (e.g., temperature, voltage, stretch, etc.), chemical factors (e.g., lipids, pH and ions), and multiple intracellular signalling messengers [8,9,10,11].

The roles of TRPV4 and TRPM8 in vascular function have been already shown in several studies, including our own work [12,13,14]. Among other members of the TRPM and TRPV subfamilies, these subtypes are the most abundant both in systemic and pulmonary arteries [15]. The authors [15] conducted experiments to determine the expression of various types of melastatin- and vanilloid-related channels in pulmonary arterial smooth muscles. Quantitative real-time RT-PCR showed that TRPV4 and TRPM8 were the most abundantly expressed TRPV and TRPM subtypes in the myocytes of the isolated pulmonary arteries [15]. TRPV4 had been initially characterised as a channel activated by cell swelling initiated by hypotonicity [16], but subsequently, many other activating stimuli were discovered: shear stress, heat, pH changes, and exogenous and endogenous agonists [17]. In contrast to TRPV1, TRPV2, and TRPV3 thermosensors, which are activated by heat above 43, 52 and 39 °C, respectively, and show sensitisation upon repeated heat stimulation [18,19,20,21,22], TRPV4 is activated by warm temperature above 27–34 °C, and it demonstrates desensitisation after repeated heating [21,23]. Nilius and co-workers reported [17] that the TRPV4 channel is constitutively active at physiological temperature and shows Ca^2+^-dependent inactivation. TRPV4 can also be activated by different ligands. The best characterised endogenous activators are endocannabinoids and arachidonic acid metabolites (5,6-EET), as well as other exogenous molecules, such as bisandrographolide A (BAA, natural product), α-phorbol esters (4α-PDD, derivative of a natural product), and GSK1016790A (synthetic) [24,25]. TRPV4 are cation channels with high permeability to Ca^2+^ and Mg^2+^ compared to Na^+^ and K^+^ [17]. TRPV4 is highly expressed in pulmonary arteries, especially in vascular smooth muscle cells [5,15,26,27] and the endothelium [23], DRG neurons [28], skeletal muscle [29], lungs [30,31], and insulin-secreting β cells of the pancreas [32].

The other TRP member, TRPM8, is known as a Ca^2+^-permeable non-selective cationic channel (P_Ca_/P_Na_~1–3.3), and polymodal sensor activated by cold temperature (8–25 °C), cooling compounds (menthol, icilin), voltage and other stimuli [33,34,35]. TRPM8 was initially cloned from prostate epithelium tissue [36] and later identified as a menthol-activated ion channel in cold-sensitive sensory neurons [34,37]. TRPM8 gene knockout mice models proved that the channel played a pivotal role in cold temperature perception [38,39]. These channels are highly expressed not only in neuronal but also in non-neuronal tissues, such as skin, oral epithelium, nasal mucosa, pulmonary tissues, bladder, male urinary and genital tracts, colon, and the vasculature [14,15,38,40,41,42,43].

TRPV4 and TRPM8 channels very often interact with other cellular signalling structures (channels and receptors), and that cooperation results in different ways of cell signalling pathway regulation. It was reported that there was a synergistic interplay of intermediate conductance calcium-activated potassium (K_Ca_3.1) channels and TRPV4 channels in lung disease and pulmonary circulation [44]. The authors found that K_Ca_3.1 channels play a role in downstream TRPV4 signal transduction and as a potential target for the prevention of unwanted vasodilatation and pulmonary circulatory collapse. Additionally, it was demonstrated that K_Ca_3.1 channels determine the proliferation phenotype of human bronchial smooth muscle cells through TRPV4 channels in chronic asthma, making it a potential therapeutic target to treat this disease [45]. In primary cultured rat mesenteric artery endothelial cells (MAECs), a unique type of heteromeric channels complex from three different TRP subfamilies was found, TRPV4-TRPC1-TRPP2 channels [46]. Thus, a prevalent role of heteromeric TRPV4-C1-P2 channels in flow-induced Ca^2+^ entry into rat MAECs has been described. The interplay of TRPV4 and TRPC1 channels has also been described [47]. The heteromeric TRPV4–C1 complex showed different electrophysiological properties compared to homomeric TRPV4 channels. TRPM8 channels were also found to function as part of the complex regulation by several receptor-coupled signalling pathways, such as PLC, iPLA_2_, M3/cPLA_2_, and α2a-ARs/Gi/AC/PKA pathways [48,49,50,51,52].

TRPV4 could be a therapeutic target for the treatment of cardiovascular pathologies. Current research shows that activation of the TRPV4 channel enhances vascular reactivity in chronic hypoxic pulmonary hypertension. However, very little is known about the properties and regulation of this channel in vascular tissue. Furthermore, TRPV4 channels are overexpressed under chronic hypoxia [6,53]. In summary, TRPV4 and TRPM8 Ca^2+^-permeable channels play an important role in the regulation of vascular tone and contractility, but their signalling pathways, interaction with other channels and receptors, and activation mechanisms are still a subject of intense investigation. Here, we focused on the partnership of vascular TRPV4 and TRPM8 channels with RyRs, BK_Ca_, and Ca_L_ channels as the three major players shaping membrane potential changes and vascular contractility. We propose that the TRPV4/M8-RyR-BK_Ca_/Ca_L_ interaction forms the basis for both their local (via Ca^2+^ sparks) and global (via membrane potential changes) interactions underlying, depending on conditions, a biphasic vascular contractile response initiated by TRP activation.

## 2. Materials and Methods

### 2.1. Cell Isolation and Tissue Preparation

Experiments were performed on pulmonary arteries (PAs) freshly dissected from 10–12-week-old Wistar male rats (200–250 g) and ventral tail arteries (TAs) from male Sprague Dawley rats (10–12 weeks). Animals were humanely euthanised via cervical dislocation or lethal I.P. injection of sodium pentobarbital (~300 mg/kg body weight). The thoracic cavity was dissected, and the cardiopulmonary complex was removed into the modified Krebs solution containing (mM): 120 NaCl, 12 glucose, 10 HEPES, 6 KCl, 2.5 CaCl_2_, 1.2 MgCl_2_, and pH 7.4 adjusted by adding NaOH. Next, the main branches of PA (extrapulmonary) were cut out from the complex and cleaned of connective and adipose tissues. Arteries were sliced into small fragments, which subsequently were enzymatically treated in (mg/mL) papain (1 mg/mL; Sigma-Aldrich, St. Louis, MO, USA), bovine serum albumin (BSA; 1 mg/mL), and dithiothreitol (1 mg/mL; Sigma-Aldrich) for 20 min, followed by the mixture of collagenase type 1A (1.5 mg/mL; Sigma-Aldrich), bovine serum albumin (1.5 mg/mL; Sigma-Aldrich), dithiothreitol (1 mg/mL) for 15 min at 36.5 °C in Ca^2+^-free Krebs solution containing (in mM) 120 NaCl, 12 glucose, 10 HEPES, 6 KCl, and pH 7.4 by NaOH. Enzymes were washed out by Ca^2+^-free Krebs solution, and isolated myocytes were released by gently triturating the thus digested tissue fragments with Pasteur pipette. Freshly isolated cells were placed onto coverslips with addition of modified Krebs solution at 1:2 ratio. SMCs were stored at 4–6 °C and were used in experiments within 6–8 h.

Dissected rat TAs were cleaned of connective tissue and cut into 1–2 mm lengths segments in the modified Krebs solution. Vascular segments were transferred to a dissociation medium (DM) containing (in mM) 110 NaCl, 5 KCl, 0.5 KH_2_PO_4_, 0.5 NaH_2_PO_4_, 10 NaHCO_3_, 10 HEPES, 10 taurine, 10 glucose, 0.5 EDTA, 2 MgCl_2_, 0.03 phenol red, 0.16 CaCl_2_, pH 7.4 (adjusted with NaOH) containing collagenase type 1A (1 mg/mL), collagenase type XI (0.5 mg/mL; Sigma-Aldrich), soybean trypsin inhibitor (1.5 mg/mL; Sigma-Aldrich), and BSA (1 mg/mL). Then, segments were incubated at 37 °C for 15–20 min. Fire polished glass pipette was used to release single cells by trituration. Cell suspension was stored at 4 °C in low-Ca^2+^ Krebs and used in electrophysiological experiments within 6–8 h.

For contraction recordings, the proximal part of the PA (0.3–0.5 cm) was taken, dissected free from surrounding connective tissues, and cut into small rings under a binocular dissection microscope. Ventral TA was removed by careful dissection and placed in modified Krebs solution at room temperature, cleaned of excess connective tissue and cut into ~4 mm size vascular rings.

All experiments involving animals were conducted in accordance with guidance of the European Convention for the Protection of Vertebrate Animals used for Experimental and other Scientific Purposes and approved by the Ethics Committee of the Ukrainian Association for the Use of Animals in Research and Education while complying with the UK Animal Scientific Procedures Act (1986) and the principles set out in the Guide for the Care and Use of Laboratory Animals (National Academy of Sciences of Ukraine, 1996).

### 2.2. Tensiometry

Vascular smooth muscle rings of PA and TA were used to study tension under action of different agents. An artery was dissected and cleaned as described before and cut into the rings with diameter of 0.9 × 1.5 mm and 1.5–2 mm wide for PA and 4 mm wide for TA. All procedures were carried out at room temperature in the modified Krebs solution. These smooth muscle samples were mounted onto the stainless-steel hooks of the tensiometric setup, with one stationary hook and one attached to an isometric force transducer. For PA experiments, the force transducer (AE 801, SensoNor A/S, Norten, Norway) was coupled to an AD converter (Lab-Trax 4/16, World Precision Instruments, Inc., Sarasota, FL, USA). The data during were recorded continuously using DataTrax 2 software (World Precision Instruments, Inc., Sarasota, FL, USA). For experiments with TA, the rings were attached to force transducers (Piodem, UF1, 25 g; Digitimer Ltd., Welwyn Garden City, UK), while responses were transmitted to the amplifier (NL108, Neurolog, Digitimer Ltd., Welwyn Garden City, Hertfordshire, UK) and recorded using a laboratory interface (Micro 1401, Cambridge Electronic Design, Cambridge, UK) and a data-acquisition software (Spike 2, Cambridge Electronic Design). The hooks were immersed in a tissue bath containing flowing modified Krebs-bicarbonate solution containing (in mM) 133 NaCl, 4.7 KCl, 16.3 NaHCO_3_, 1.38 NaH_2_PO_4_, 2.5 CaCl_2_, 1.2 MgCl_2_, 10 HEPES, 7.8 D-glucose, and pH adjusted to 7.4 with NaOH and kept at 37 °C. Resting tension of PA rings was adjusted to 0.5 g and for TA to 0.75 g. Samples were allowed to equilibrate for 1 h under resting tension before the experiments; then, high-potassium solution (60 mM KCl) was applied into the bath in order to stimulate contractile activity, thus evaluating the functional state of the tissue. Contractile activity of vascular rings was stimulated by the application of an agonist of alpha-adrenoceptor (α-AR) and phenylephrine (PhE, 2 or 10 µM). In some experiments, vascular rings endothelium-denuded with saponin (PA) or by passing a fine wire down the vessel lumen (TA) were used as previously detailed, including the acetylcholine test for successful removal of the endothelium [12,14]. In brief, the standard acetylcholine test was conducted to confirm successful de-endothelisation of blood vessels with the saponin solution. De-endothelisation control is a well-known test using acetylcholine, which causes distinct vasodilatation in tissue with intact endothelium, whereas, in de-endothelised vessels, it causes vasoconstriction. In our experiments, application of acetylcholine (10^−5^ M) caused vasoconstriction, confirming the full de-endothelisation of vessels.

### 2.3. Patch-Clamp Recordings

Spontaneous transient outward membrane current (STOC) recordings were made using standard whole-cell patch-clamp techniques at room temperature (20–22 °C) from dispersed single smooth muscle cells with modified Krebs as the external solution. The intracellular solution contained (in mM): 130 KCl, 1 MgATP, 5 creatine, 10 glucose, 0.3 EGTA, and 10 HEPES (pH adjusted to 7.4 with KOH). Membrane potential changes were recorded in the current-clamp mode using a gap-free protocol with the internal and external solutions, as described above. Thick-walled patch pipettes were made from borosilicate glass (1.5 mm OD, 0.86 mm ID; Harvard Apparatus, Holliston, MA, USA) using a P-97 Flaming/Brown micropipette puller (Sutter Instruments, Novato, CA, USA) and when filled with solution and had a resistance of approximately 3–4 MΏ. Initially, a tight seal between the pipette and cell membrane was formed, then fast capacitive transients were minimised, and, providing the input resistance exceeded 1 GΏ, further patch-clamp recordings were conducted. Cell membrane capacitance and series resistance were compensated electronically (the latter by 50–70%) using relevant amplifier controls. Patch-clamp electrophysiology was performed using an Axopatch 200B voltage-clamp amplifier (Molecular Devices, Sunnyvale, CA, USA). Low-pass filtering was set to 2 kHz and 0.2 kHz for current clamp and STOCs recordings, respectively; data were digitised at 10 kHz with a Digidata 1322A interfaced to a computer running the pClamp 8 program (Molecular Devices, Sunnyvale, CA, USA).

### 2.4. Intracellular Calcium Recordings

Ratiometric [Ca^2+^]_i_ recordings were conducted using photometric microscopy in fura-2 AM (Molecular Probes, Invitrogen, Carlsbad, CA, USA) loaded myocytes. To facilitate Fura-2 loading, 2.5 mg/mL pluronic acid (Sigma-Aldrich), diluted in 5 μL DMSO, was also added to the solution. Standard excitation (340 and 380 nm) and emission (510 nm) wavelengths were used. Other details of [Ca^2+^]_i_ recordings can be found elsewhere [13].

### 2.5. Statistical Analysis

Recordings were analysed and plotted using Clampfit 10 (Molecular Devices, Sunnyvale, CA, USA) and Origin 9.5 software (OriginLab Corporation, Northampton, MA, USA). Data are shown as means ± S.E.M., where *n* signifies the number of tested arterial rings or isolated cells taken from N animals. The Kolmogorov–Smirnov test was used to verify that the data is normally distributed, followed by Student’s two-tail *t*-test and, where appropriate (three or more sets of data) one-way analysis of variance (ANOVA) with either Tukey’s (for all pairs of data sets) or Bonferroni’s (for selected pairs of data) post hoc test for statistical comparisons. Differences at *p* < 0.05 were considered to be significant.

## 3. Results

### 3.1. Contractile Responses to TRPV4 and TRPM8 Activation

#### 3.1.1. The Biphasic Reaction of Pulmonary Artery via TRPV4-Channels after α-Adrenoceptors Activation

**Figure 1:**
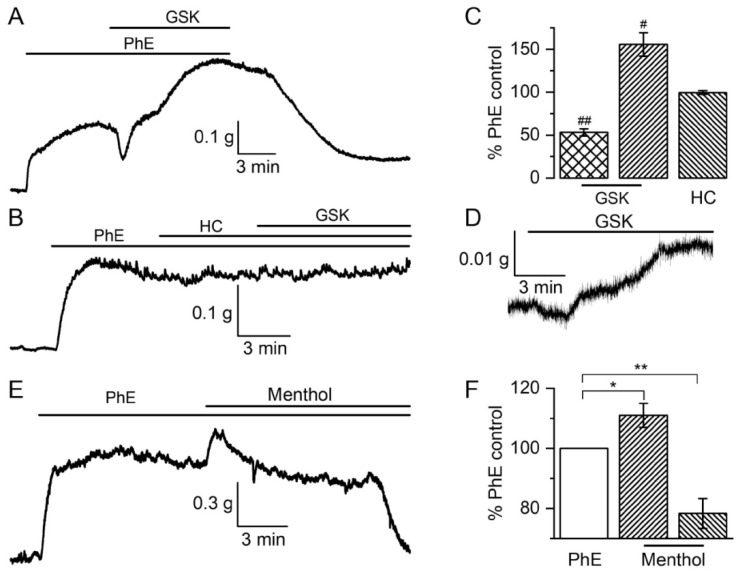
The biphasic response of TRPV4 (**A**–**D**) and TRPM8 channels (**E**,**F**) activation in VSM preconstricted with phenylephrine. (**A**) Representative tensiogram showing biphasic contractile response to GSK1016790A (0.3 μM) consisting of transient relaxation and large contraction in PA ring pre-constricted with PhE (10 μM). (**B**) Selective blocker of TRPV4 channels, HC067047 (1 μM), inhibits the biphasic response to GSK1016790A. (**C**) Comparison of GSK1016790A (*n* = 20, N = 12) and HC067047 (*n* = 4, N = 2) action; data are percentage ratios (mean ± SEM) relative to the peak of the PhE-induced response. ^#^—*p* < 0.001, ^##^—*p* < 0.0001 compared to 100% by one sample *t*-test. (**D**) GSK1016790A caused very small relaxation and slight contraction when PA was not pre-treated with PhE (*n* = 6, N = 3). (**E**) Representative tensiometric trace illustrating the biphasic effects of menthol (300 μM) on a PhE-constricted (2 μM) TA vascular ring. (**F**) Summary data quantifying the effects of menthol, menthol peak and late (after 10 min) values as percentage of the PhE-induced response before menthol application. * *p* < 0.05, ** *p* < 0.01 compared to 100% by one sample *t*-test (*n* = 9, N = 9). In all these experiments, pulmonary artery rings with intact endothelium were used.

In our functional experiments, the action of the TRPV4 agonist GSK1016790A on PA was studied using isometric contractile measurement. To provide more physiologically relevant conditions, the vessels were pre-activated by an adrenergic receptor agonist, PhE (10 μM). Surprisingly, the application of 0.3 μM GSK1016790A caused a rather complex biphasic response: fast relaxation to 53 ± 4% (all values here and below are calculated as percentage ratios relative to the peak of the PhE-induced response) was followed by strong and slow contraction to 156 ± 14% (*p* < 0.001 compared to 100% by *t*-test in both cases, *n* = 20, N = 12, Figure 1A,C). In prior tests, 50 nM GSK had no or very little effect on PhE-induced contractions. The involvement of TRPV4 channels in this response was evidenced by the observation that both phases were abolished by their selective inhibitor, HC067047 (1 μM), to 95 ± 3% (*p* = 0.19) and 99 ± 2% (*p* = 0.65), respectively (compared to 100% by *t*-test in both cases; *n* = 4, N = 2, Figure 1B,C). Activation of α-ARs had a key role in the development of such a response to GSK1016790A because its application at the basal tension caused very little relaxation followed by only a small contraction phase to 22 ± 4% (*p* < 0.0001, *n* = 6, N = 3, Figure 1D). Additionally, it should be noted that GSK1016790A application in high potassium solution (60 mM KCl) did not produce any effect (data not shown, *n* = 3), which additionally confirms the importance of TRPV4 channels (and its other partner channels as will be shown later), and not of the pre-contraction conditions, for the development of the complex biphasic PA contractile response.

#### 3.1.2. Responses of Tail Artery to TRPM8 Agonist Menthol

In our previous study, TRPM8 involvement in basal vascular tone responses was presented in tensiometric experiments on rat TA vascular rings [13,14]. The application of the TRPM8 agonist menthol (from 100 to 500 μM) to the bath induced dose-dependent vasoconstriction. These data indicated that menthol was able to induce direct vascular responses even in the absence of the Ca^2+^ mobilising effects of PhE. The application of menthol (300 μM) induced additional contraction followed by vasodilatation of vascular rings preconstricted by PhE (2 μM) (Figure 1E,F). However, it should be noted that the interpretation of these results was complicated by the fact that menthol at this concentration, unlike GSK1016790A in the case of TRPV4, also significantly inhibits Ca_L_ channels [13].

#### 3.1.3. The Involvement of Other Channels in the Biphasic Action of GSK1016790A

**Figure 2:**
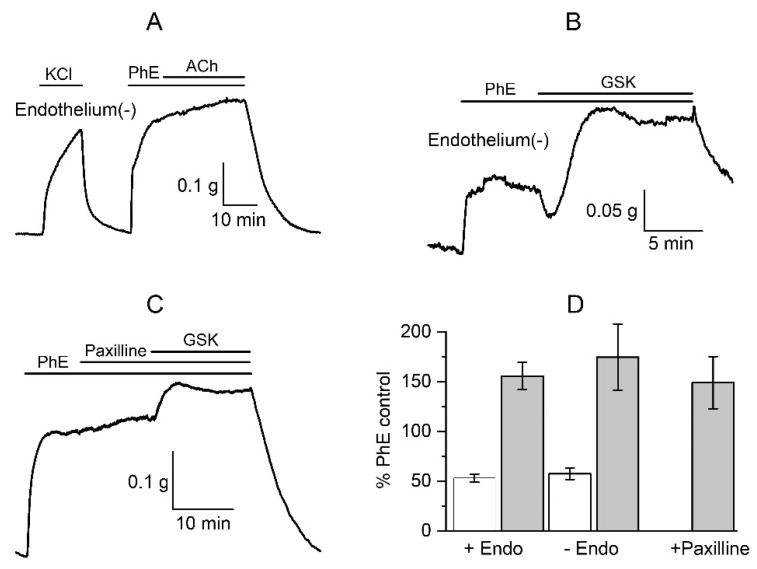
BK_Ca_ channels are involved in the biphasic response due to the activation of TRPV4 channels in PA SM. (**A**) The removal of endothelium was confirmed by the acetylcholine (ACh, applied at 10 µM) test. (**B**) The potential role of endothelial TRPV4 channels is insignificant in endothelium-denuded PA response to GSK1016790A. (**C**) The selective BK_Ca_ channel inhibitor, paxilline (500 nM), abolishes the relaxation phase of the biphasic response to GSK1016790A (0.3 μM) in intact PASM pre-contracted with PhE (10 μM). (**D**) Data are presented for the relaxation (white columns) and contraction (grey columns) phases of GSK1016790A-induced responses as percentage of the PhE-induced response in intact (+Endo, *n* = 20, N = 12) and endothelium-denuded (−Endo, *n* = 5, N = 3) samples. In the presence of paxilline, only the contraction phase developed (*n* = 10, N = 5). ANOVA with Tukey’s post hoc test showed no statistically significant differences in either relaxation (+Endo vs. −Endo) or contraction responses (all 3 data sets) under these various conditions.

Given the complex response of PA to the TRPV4 agonist, the next task was to find out the mechanisms involved. It was shown that TRPV4 channels are highly expressed in vessel endothelium, and activation of endothelial TRPV4 evokes vasodilatation [54]. Thus, it could be assumed that the relaxation phase was due to the activation of endothelial TRPV4 channels. However, we found that endothelium-denuded arteries, as confirmed using the acetylcholine test (Figure 2A), responded the same way as intact vessels. In vessels pre-contracted with PhE, GSK1016790A caused relaxation to 57 ± 6% followed by contraction to 175 ± 33% (*n* = 5, N = 3), which was only slightly higher (but not statistically significantly, *p* = 0.56) in endothelium-denuded samples compared to intact tissue (Figure 2B,D). These results indicate the involvement of smooth muscle TRPV4 channels rather than endothelial ones in the development of biphasic reactions of the PA.

Large-conductance Ca^2+^-activated potassium channels (BK_Ca_) are the most likely partner channel contributors to this complex response. Earley et al. [5] described vasorelaxation of a cerebral artery in response to epoxyeicosatrienoic acids (EETs, endothelium-derived activators of TRPV4-channels) due to BK_Ca_ channels activation that occurs as a result of Ca^2+^ release from the SR. In this study, we demonstrate that the BK_Ca_ channels inhibitor, paxilline (500 nM), prevented the development of the first vasorelaxation phase (96 ± 1%, *p* < 0.001 if compared to relaxation to 53 ± 4% without paxilline) during the GSK1016790A-induced reaction, while still increasing the vascular tone to 149 ± 26% (*n* = 10, N = 5), indicating that BK_Ca_ channels are indeed involved in the biphasic reaction of PAs (Figure 2C,D).

**Figure 3:**
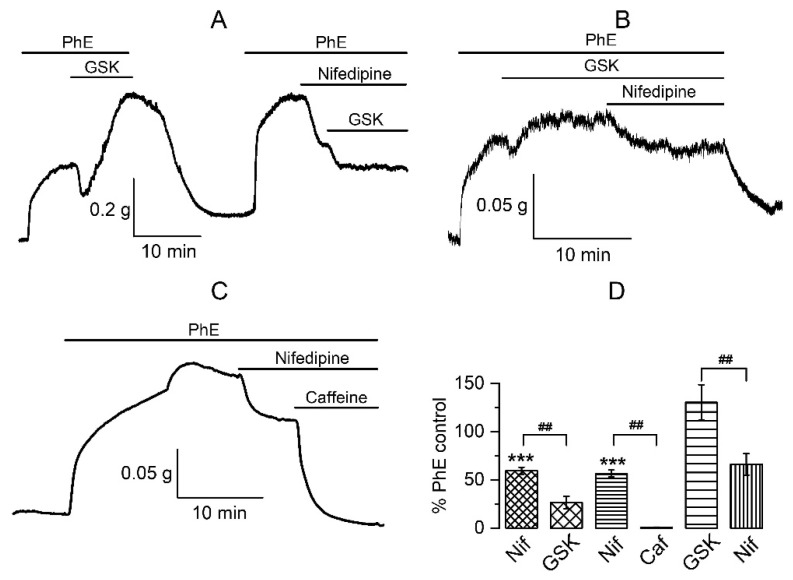
The contraction phase of the biphasic response is sensitive to the calcium channels’ blocker and may involve the RyRs-dependent signalling pathways. (**A**) The selective blocker of Ca_L_, nifedipine (10 μM), prevents the development of contraction phase in response to GSK1016790A (0.3 μM) while accentuating the relaxation phase. (**B**) The contraction phase induced by GSK1016790A is inhibited by nifedipine. (**C**) Caffeine (10 mM) acts the same way as GSK1016790A in PA pretreated with PhE and nifedipine. (**D**) Comparative analysis of GSK1016790A (*n* = 6, N = 3) and caffeine (*n* = 4, N = 2) action on intact PA SM pretreated with PhE and nifedipine, as well as nifedipine effect on intact PA SM pretreated with PhE and GSK1016790A (*n* = 5, N = 4). Data are presented as percentage of the PhE-induced response (mean ± SEM). *** *p* < 0.0001 compared to 100% by 1 sample *t*-test; ## *p* < 0.001 by ANOVA with Tukey’s post hoc test. In all these experiments, pulmonary artery rings with intact endothelium were used.

We have already described that the contractile phase was due to Ca^2+^ entry. In a Ca^2+^-free solution, relaxation was retained in response to GSK1016790A, whereas the contraction was abolished [12]. An important source of calcium in VSM is provided by openings of L-type voltage-gated Ca^2+^ channels (Ca_L_). Thus, we next investigated the functional outcomes of TRPV4 activation while blocking Ca_L_. Application of nifedipine (10 μM) evoked the expected relaxation of smooth muscle to 59 ± 4% (*p* < 0.001 compared to 100%), while subsequent addition of GSK1016790A caused large and sustained vasodilatation to 27 ± 6% (*n* = 6, N = 3), as opposed to its normal action initiating significant contraction (Figure 3A,D). In a complementary series of experiments, nifedipine (10 μM), when applied during the GSK1016790A-induced contraction phase, caused relaxation from 130 ± 18% to 66 ± 11% (*n* = 5, N = 4) (Figure 3B,D). These results, for the first time, revealed another important participant in the multicomponent response to TRPV4-channels activation, Ca_L_. Inhibition of these channels prevents the development of vasoconstriction and even promotes the relaxation phase making it more sustained. This can be due to a combination of several events, calcium channel blockade, membrane hyperpolarisation, and depletion of Ca^2+^ stores.

Involvement of the Ca^2+^ store in the TRPV4-initiated response was previously shown in other types of vessels, where the key role of RyRs was indicated [5,6]. Our data regarding Ca_L_, BK_Ca_, and TRPV4 channel communication could also be explained by Ca^2+^ store involvement in this process. The effects of GSK1016790A in the presence of nifedipine in PASM are also, for the most part, analogous to those produced by caffeine, which causes Ca^2+^ release [55,56]. Thus, caffeine (10 mM) evoked significant additional vasodilatation to 8 ± 1% of the PhE-induced response (Figure 3C,D) even after the tone had been already reduced by nifedipine to 57 ± 4% (*n* = 4, N = 2). These results suggest that both agonists of TRPV4 channels and caffeine have the same target. Interestingly, GSK1016790A application after the combined action of nifedipine and caffeine did not produce any effect, neither contraction nor relaxation (e.g., the amplitude of contraction remained at 9 ± 1% (*n* = 4) of the PhE-induced response in control).

### 3.2. Membrane Potential Recordings

#### 3.2.1. The Effect of TRPV4 Activation by GSK1016790A on Membrane Potential in Pulmonary Artery Vascular Smooth Muscles Cells (VSMCs)

**Figure 4:**
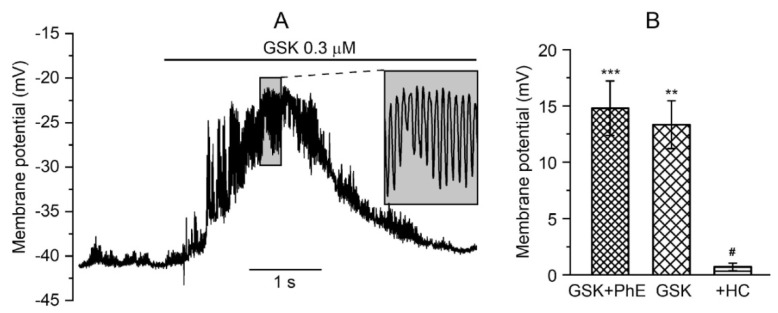
TRPV4-mediated changes in membrane potential in current-clamped PA myocytes. (**A**) At membrane potentials in the physiological range for VSMCs (−40 to −50 mV), GSK1016790A induces a slowly developing depolarisation of the membrane and simultaneously causes an increase in the frequency of spontaneous hyperpolarisation events. (**B**) These effects were abolished in the presence of HC067047 as an independent test for specific involvement of TRPV4. ** *p* < 0.01, *** *p* < 0.001 compared to 0 by 1 sample *t*-test; ^#^ *p* < 0.01 by ANOVA with Tukey’s post hoc test compared to the GSK data.

Membrane potential is an important unifying factor defining ion flux via all plasmalemmal ion channels since current magnitude is proportional to the electrochemical gradient for ions, especially via voltage-gated channels, which show additional dependences of their open probability (Po) on membrane potential. In any individual myocyte, the plasma membrane is an equipotential surface; hence, membrane depolarisation due to TRP channel opening will affect all other voltage-dependent channels, thus providing a mechanism for distant global signalling. Thus, Ca^2+^ spark-activated BK_Ca_ channels produce hyperpolarising current, which counteracts vasoconstriction induced by PhE and GSK and, in turn, regulates pulmonary vascular tone. Figure 4A shows that after the application of PhE and GSK1016790A (*n* = 12, N = 7), slow depolarisation developed, on which high-frequency spontaneous hyperpolarisation events were superimposed, most likely due to the activity of BK_Ca_ channels forming STOCs [55,57].

Mean membrane potential changes are shown in Figure 4B. In five cells, to which only GSK1016790A was applied, it caused an increase in membrane potential of 13.3 ± 2.1 mV (*n* = 5, N = 3) from the resting membrane potential in the physiological range from −40 mV to −50 mV. Membrane depolarisation of 14.8 ± 2.4 mV (*n* = 7, N = 4) was observed when PhE and GSK1016790A were co-applied, while in the presence of the TRPV4 blocker HC067047, changes in membrane potential were insignificant, only 0.7 ± 0.3 mV (*n* = 5, N = 3).

Thus, GSK1016790A appears to trigger cation influx across the plasma membrane, causing membrane depolarisation and activation of Ca_L_, while, at the same time, this potentiates Ca^2+^ spark events, as indicated by the superimposed spontaneous hyperpolarisations.

#### 3.2.2. Membrane Potential Responses to TRPM8 Agonist Menthol in Tail Artery Myocytes

**Figure 5:**
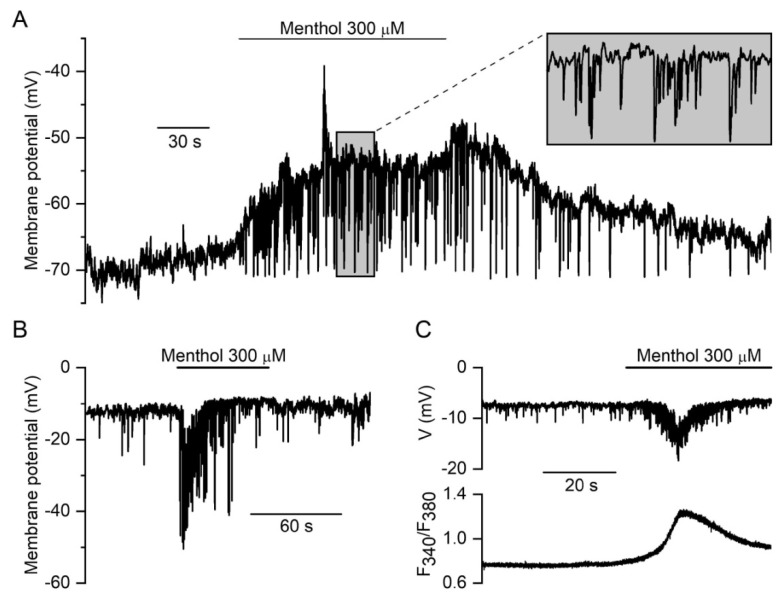
Menthol-induced changes in membrane potential in a current-clamped rat TA myocytes. (**A**) At membrane potentials in the physiological range for VSMCs (−45 to −65 mV), menthol (300 μM) causes slow membrane depolarisation accompanied by pronounced spontaneous hyperpolarisation events (**B**,**C**). In contrast, in depolarised cells, only high-frequency spontaneous hyperpolarisations further promoted by menthol were observed. These correlated with an increase in [Ca^2+^]_i_ measured in the same cell (**C**).

The effect of menthol (300 μM) on cell membrane potential in a representative current-clamped rat tail artery myocyte with a resting membrane potential of about −65 mV, which is within the normal physiological range for this cell type [58], is illustrated in Figure 5A. Application of menthol caused a slowly developing depolarisation of 8.3 ± 1.1 mV from an initial potential of −56.8 ± 3.1 mV (*p* < 0.001, Student’s paired *t*-test, *n* = 7, N = 5), which was accompanied by accelerated spontaneous hyperpolarisation events, which are caused, as already discussed, by Ca^2+^ sparks triggering STOCs. Although not investigated in detail, in our previous work, Ca^2+^ sparks augmented by menthol have indeed been evident in rat TA myocytes (see Figure 7D in [13]). These spontaneous hyperpolarisation events increased in frequency from 0.3 ± 0.1 Hz measured in the 30 s prior to menthol application to 1.4 ± 0.4 Hz in the 30 s afterwards (*p* < 0.05; Student’s paired *t*-test, *n* = 7, N = 5). This acceleration of ×4.7 corresponded with increasing in STOC frequency of ×3.6 after menthol application, as will be illustrated later.

In NSCCs such as TRPs, currents typically reverse at potentials in the range from −10 to 0 mV. Interestingly, in a few atypically depolarised cells with resting potentials greater than −20 mV (*n* = 4), menthol produced only an increase in the amplitude and frequency of spontaneous hyperpolarisations. These were associated with an increase in [Ca^2+^]_i_ measured in the same cell (Figure 5B,C). These findings directly confirm the activation of non-selective cation conductance (e.g., current with a reversal potential of about −10 mV), rather than a decrease in potassium conductance, as the cause of membrane depolarisation.

Since the rate of membrane potential change (dV/dt) is directly proportional to net transmembrane current amplitude I_M_ (and inversely proportional to cell capacitance C_M_), i.e., dV/dt = I_M_/C_M_, the slow development of membrane depolarisation in response to menthol implies that the net inward current producing it is very small. In fact, taking into account the C_M_ value of 30.2 ± 1.5 pF (*n* = 7) from the parameters of depolarisations in current-clamped TA myocytes, we have estimated the maximal net inward current as 0.037 ± 0.006 pA (*n* = 7, N = 5). Similar estimates in the case of PA and GSK action revealed somewhat larger, but still in the pA range, maximal net inward current (~0.6 pA) producing slow membrane depolarisation (Figure 4A).

### 3.3. Effects of TRPM8 and TRPV4 Activation on Ca^2+^-Activated Membrane Currents in Vascular SMCs

**Figure 6:**
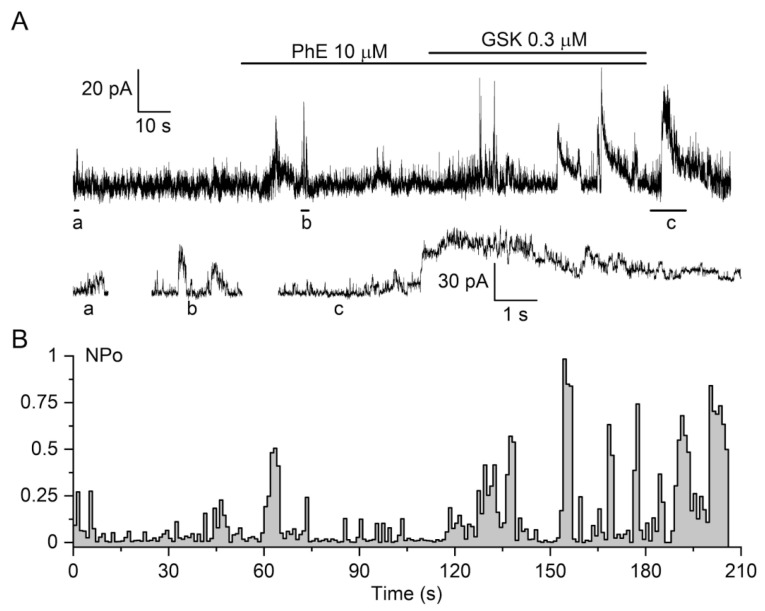
TRPV4 agonist induces an increase in the frequency of STOCs in isolated PA myocytes. (**A**) Representative whole-cell recording from PA myocyte held at −20 mV (expended trace segment denoted as “a”) (*n* = 6, N = 4). Application of PhE caused initiation of larger STOCs (b), whereas GSK1016790A (0.3 μM) further enhanced generation of STOCs (c). (**B**) Corresponding BK_Ca_ channel activity quantified in terms of channel open probability (NP_O_).

As already noted, both tensiometric and current clamp experiments strongly implied cross-talk between TRPV4 and Ca^2+^-activated BK_Ca_ channels, the latter being activated by local rises in [Ca^2+^]_i_. While GSK1016790A activated TRPV4 channels and induced Ca^2+^ release from Ca stores, we next asked whether or not it indeed potentiated STOCs discharge as a functional correlate of localised Ca^2+^ release from a cluster of RyRs, or Ca^2+^ sparks [55,57,59]. STOCs were recorded in voltage-clamped rat PA VSMCs in the presence of PhE and GSK1016790A. Figure 6A illustrates a representative whole-cell recording showing that the activation of TRPV4 by GSK1016790A (0.3 μM) increased the frequency of STOCs, most likely due to cross-talk between TRPV4 and BK_Ca_ channels, as was previously described in cerebral artery smooth muscle cells [5]. Notably, BK_Ca_ channel density and/or activity is rather low in PA myocytes as single-channel events are resolvable even in the whole-cell configuration, as can be more clearly seen in current trace segments on an expended time scale (Figure 6A, bottom). This allowed us to quantify BK_Ca_ channel activity in terms of NP_O_ (Figure 6B), showing periodic several-fold increases in NP_O_, which were induced by PhE and further potentiated by the TRPV4 agonist (Figure 6B).

**Figure 7:**
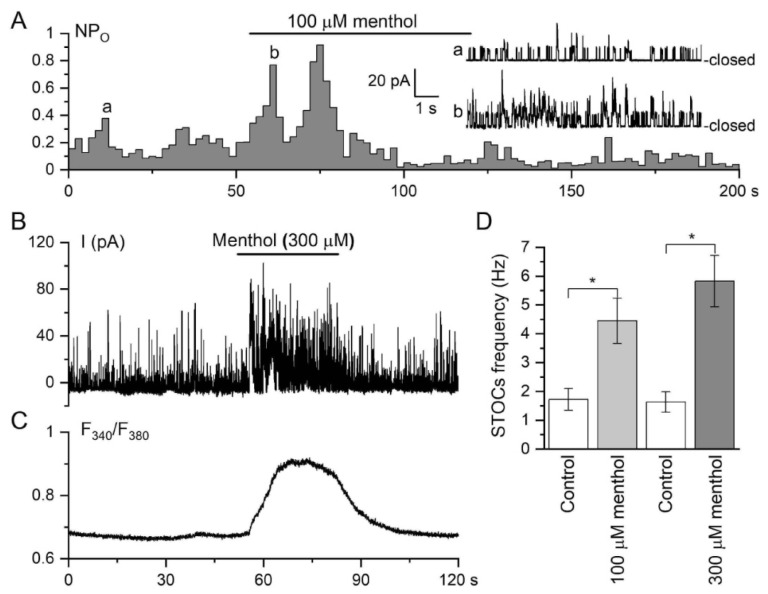
TRPM8 agonist menthol increases frequency of STOCs in an isolated TA myocyte by activating clusters of BK_Ca_ channels. Inset shows trace segments denoted as *a* and *b* on an expanded time scale. (**A**) Formation of STOCs results from simultaneous openings of several BK_Ca_ channels seen as periodic increases in their NPo values when recorded in the cell-attached patches held at 140 mV relative to cell resting potential. (**B**) Representative whole-cell recordings from a TA VSMC held at −20 mV (*n* = 8). The Fura-2 loaded cell was recorded in the absence and presence of the TRPM8 agonist menthol applied at 300 μM. (**C**) Note that menthol-induced increases in STOCs frequency coincide with a rise in [Ca^2+^]_I_ recorded in the same myocyte. (**D**) Mean frequency of STOCs discharge in control (white columns, *n* = 6–7) and after menthol applications at concentrations indicated. * *p* < 0.01 by Student’s paired *t*-test.

In contrast, BK_Ca_ channel density and/or activity were much higher in TA myocytes. In the cell-attached configuration, if the pipette was by chance located near a region with a high probability of Ca^2+^ spark discharge (termed frequent discharge sites (FDSs) [2,59]), it was even possible to see how STOCs occurred as a result of simultaneous activation of several BK_Ca_ channels, which became especially evident after menthol application (Figure 7A). In the whole-cell configuration, menthol augmented STOCs (Figure 7B). Additionally, 100 μM menthol induced a significant increase in the frequency of STOC events from 1.72 ± 0.38 to 4.45 ± 0.78 Hz (*n* = 6, N = 4), while 300 μM menthol induced a further increase from 1.63 ± 0.35 to 5.83 ± 0.89 Hz (*p* < 0.01; *n* = 7, N = 5; Figure 7D).

These electrophysiological findings suggested that the effects of both menthol and GSK1016790A on Ca^2+^-dependent plasma membrane conductances were the result of TRP agonist-induced discrete Ca^2+^-release events, which were translated into STOCs activity. Nevertheless, it is important to note that Ca^2+^ entry can also strongly potentiate Ca^2+^ spark activity; thus, Ca^2+^ release and influx could both be involved in the underlying mechanisms of TRPM8 and TRPV4 actions in the vasculature. As described above (Figure 1), activation of TRPV4 induced a significant but short-lasting relaxation, which was thus caused by the activation of BK_Ca_ channels due to CICR and rises in subsarcolemmal [Ca^2+^]_i_ (Ca^2+^ sparks), as schematically illustrated in our Graphical Abstract.

## 4. Discussion

TRP channels, when activated, produce the two main functionally relevant outcomes: membrane depolarisation by cation influx into the cell and an increase in [Ca^2+^]_i_ due to Ca^2+^ entry and/or Ca^2+^ release. Ca^2+^ entry can be via TRP channels themselves, via Ca_L_ channels opened secondary to membrane depolarisation, as well as via Na^+^/Ca^2+^ exchange operating in reverse mode if TRP activity induces a sufficiently large local increase in the intracellular Na^+^ concentration coupled with membrane potential change [60,61]. The role of TRPV4 channels in the endoplasmic reticulum Ca^2+^ homeostasis is especially well documented [62], while Ca^2+^ entry via plasmalemmal TRP channels can also activate the classical SR Ca^2+^ channels and RyRs and InsP_3_ receptors. Regarding TRPM8 channels, our previous study has already highlighted the major role of SR-localised TRPM8 in TA myocytes [13].

**Figure 8:**
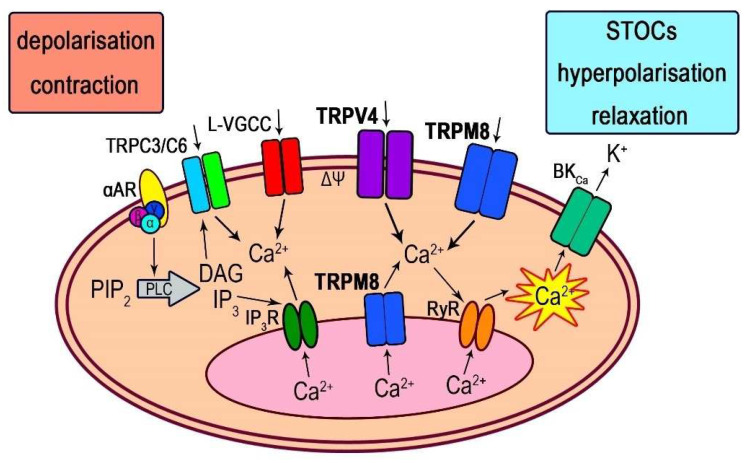
Schematic representation of TRPV4 and TRPM8 signalling pathways relevant to the present study. Intracellular Ca^2+^ is released from the SR via the activation of two types of receptors/channels: IP_3_Rs and RyRs. GSK1016790A activates TRPV4 channels, causing an influx of Ca^2+^ and Na^+^ in PA myocytes. TRPV4 can induce Ca^2+^ release from the SR via stimulation of RyRs. At the same time, α-ARs, when activated by PhE, act through the G_q/11_ signalling pathway, resulting in IP_3_-mediated Ca^2+^ release from the SR. Ca^2+^ sparks activate closely located BK_Ca_ channels, causing STOCs, membrane hyperpolarisation and, finally, vasorelaxation. Simultaneously with these events, the openings of TRPV4 channels depolarise the membrane, thus inducing voltage-dependent activation of Ca_L_ (not excluding the possible involvement of some other signalling pathways, such as store-operated Ca^2+^ entry (SOCE), the α1-AR-TRPC3/TRPC6 complex, etc.), which results in vessel depolarisation and contraction. Menthol activates SR and plasma membrane TRPM8 channels, initiating both Ca^2+^ release and Ca^2+^ influx in SM, as was shown in our previous study [13].

Thus, TRP channels are capable of engaging various other ion channels during the formation of TRP-mediated functional responses via both changes in membrane potential and/or intracellular Ca^2+^ concentration. TRPV4 is particularly well known for its ability to orchestrate the activity of multiple types of other ion channels [25]. In this context, our main findings can be summarised as follows: First, TRPV4 channels are functionally expressed in extrapulmonary arteries SMCs. Second, the vascular response in vessels pre-treated with PhE to the selective agonist of TRPV4-channels, GSK1016790A, is a biphasic reaction consisting of an initial transient relaxation followed by large, sustained contraction. This itself is a novel finding as PhE applied at submaximal concentrations (as used here) was, until now, believed to induce nearly maximally possible constriction of vascular smooth muscles. Third, the BK_Ca_ channel blocker, paxilline, inhibited the relaxation phase, and activation of TRPV4 channels initiated STOCs as well as slow membrane depolarisation with superimposed frequent hyperpolarisation events. This is also a novel type of response, as, until now, receptors were generally classified into excitatory (those causing membrane depolarisation) and inhibitory (those causing membrane hyperpolarisation). To our knowledge, a mixed response has not yet been reported. Fourth, the contraction phase was sensitive to both Ca_L_ blocker nifedipine and Ca^2+^ store depletion by caffeine. Taken together, these results indicate that TRPV4/RyR/BK_Ca_/Ca_L_ channels are coupled functionally in extrapulmonary arteries SMCs resulting in complex biphasic response, which could be physiologically important for fine tuning of vascular tone. While the role of the TRPV4/RyR/BK_Ca_ “local” arm of TRPV4-mediated signalling in vascular function is well established [63], here, we suggest a more comprehensive mechanism that includes “global” distant signalling, driven by changes in membrane potential that affect both BK_Ca_ and Ca_L_ channels (Figure 8 and Graphical Abstract).

VSMCs typically do not discharge action potentials, but under artificial conditions, when K^+^ channels are inhibited by tetraethylammonium (TEA) and Ca_L_ are activated by BayK 8644, propagating action potentials can be induced in rat mesenteric arteries [64]. Thus, the activity of K^+^ channels safeguards arteries from action potential generation that could cause detrimental vasospasm. These same channels oppose membrane depolarisation caused by the openings of TRP channels. As our estimates showed, the net inward current that drives TRPV4- or TRPM8-induced depolarisations is in the sub-pA range. Such a precarious balance of inward and outward currents implies that it can be easily compromised by up- or down-regulation of TRP channels under some pathological conditions.

These considerations highlight the role of TRP channels in health and disease. Indeed, there is currently much attention to the role of TRPV4 channels in hypoxic pulmonary vasoconstriction, chronic hypoxic pulmonary hypertension, and inflammation, including in the framework of cross-talk between TRPV4 and RyR [6,53,65,66,67,68,69,70]. With regard to TRPM8, its activation by cold is obviously limited to the cutaneous vessels and vessels close to the airway, especially in the upper airways. However, as we found in other cell types, at physiological temperature, TRPM8 can be directly activated by lysophospholipids [49], which are increasingly implicated in various pathological states, such as inflammation [71].

When TRPV4 channels are opened, extracellular calcium ions enter the vascular cell leading to calcium-induced calcium release (CICR) via RyRs of the SR. Depending on the localisation of the SR, RyRs activation results in either contraction or relaxation of SM. RyRs from sub-membrane SR generate calcium sparks, which mainly activate BK_Ca_ channels leading to transient membrane hyperpolarisation and vasorelaxation [57]. When SR is localised in the central region, then activation of RyRs leads to an increase in intracellular calcium throughout the cytoplasm and, particularly, near the contractile apparatus of the cell, which in turn leads to SM contraction. Several studies [6,15,72] have shown that in the intrapulmonary arteries the second type of RyRs (RyR2) is expressed abundantly in the SR, which is localised in the central region, whereas in the systemic vessels, the types 1 and 3 RyRs (RyR1, RyR3) are mainly present in the SR localised in sub-membranous areas and near the nucleus, respectively. Such tissue specificity of localisation can be responsible for the different SM reactions to the activation of TRPV4 and TRPM8 channels. Thus, it was demonstrated that the TRPV4-channels activators, 11-, 12-EET as well as 4α-PDD, caused relaxation of cerebral [5] and mesenteric arteries [73], whereas, in contrast, they caused constriction of intrapulmonary arteries [6].

In our extrapulmonary artery experiments, we observed a biphasic response to TRPV4 channel activation, which indicated even more complicated underlying mechanisms (Figure 1). We assume the key role in this reaction belongs to α-ARs, which induce Ca^2+^ release from the SR via inositol trisphosphate receptors (IP_3_Rs), resulting in vasoconstriction. Without PhE pre-constriction of PA, the response was much reduced and consisted of very slight relaxation and a contraction phase that was more than five times smaller (Figure 1D). Intriguingly, another group has demonstrated that application of TRPV4 channel agonist, GSK1016790A, to pre-constricted (PhE) rat PA rings caused only significant concentration-dependent vasodilatation (>80%) without the following vasoconstriction phase [74]. It was explained as endothelium-dependent relaxation due to a major contribution of nitric oxide (NO)/soluble guanylate cyclase (sGC) pathway mediated by the TRPV4 channel. Moreover, endothelium-denuded arteries lacked the relaxation response to the agonist but showed no detectable contraction. PA vasodilatation was strongly reduced by the selective TRPV4 channel antagonist HC067047. Our results show that GSK1016790A slightly increases (by ∼20%) PA basal tone, but there was a much larger response compared to the endothelium-intact vessels contraction data (∼5%) in this other study [74].

We have previously suggested [12] an explanation of biphasic reaction by PhE-initiated Ca^2+^-store depletion at the time of TRPV4-channels activation, which could result in the failure of CICR via RyRs. However, Ca^2+^ entering through TRPV4 channels could activate BK_Ca_ directly, causing vasorelaxation. Gradually the SR is filled by the downstream mechanism of sarco/endoplasmic reticulum calcium ATPase (SERCA), so Ca^2+^ entering through TRPV4 channels could initiate a contraction phase by both Ca^2+^ entry via TRPV4-channels and Ca^2+^ release from the store via RyRs.

In this study, we show that the BK_Ca_ blocker, paxilline, abolish the initial transient relaxation phase (Figure 2) and that activation of TRPV4 channels causes acceleration of STOCs (Figure 6). These results correlate well with the TRPV4-RyR-BK_Ca_ signaloplex described by Earley and co-authors [5]. According to their hypothesis, TRPV4 channels in cerebral arteries form a signalling complex with RyR and BK_Ca_ channels to provide vasodilatation via the CICR mechanism as a response to endothelial-derived factors, such as EETs. Additionally, the authors revealed a TRPV4-dependent increase in Ca^2+^ sparks and STOCs frequency via Ca^2+^ ions entry but not via Ca_L_. Thus, in the presence of a Ca_L_ blocker, diltiazem, the TRPV4-channel agonist enhanced the activity of Ca^2+^ sparks and STOCs when external Ca^2+^ was maintained at physiological levels (2 mM), while there was no difference when extracellular Ca^2+^ concentration was reduced to 10 μM. This further confirms that there are no other sources of Ca^2+^ for the development of the vasorelaxation reaction other than its entry through TRPV4 channels and release from the Ca^2+^ store. Our data indicate that STOCs occurred in PA SMCs pre-treated with PhE. This implies that RyRs were involved in this process and that calcium stores were not completely depleted, which is not in line with the previous hypothesis. BK_Ca_ channels could also be opened due to Ca^2+^ entry via TRPV4-channels, but the presence of STOCs is strong evidence favouring their activation indirectly through RyRs and Ca^2+^ sparks. Such results suggest that a mixed scenario characteristic of both types of vessels, systemic and pulmonary, is being implemented in the extrapulmonary arteries.

In our experiments, TRPV4 channels were activated using the synthetic agonist GSK1016790A [75,76], which does not affect TRPM8 channels. Currently, GSK1016790A is one of the most commonly used TRPV4 agonists. Many authors in their studies used the same concentration of GSK1016790A (0.3 µM) [77,78] or even a higher dose [79]. It was shown that GSK1016790A (0.1–1000 nM) evokes Ca^2+^ influx in mouse and human TRPV4-expressing human embryonic kidney (HEK) cells [75]. With regard to TRPV4 antagonists, HC 067047 and RN-1734 were the first TRPV4 selective antagonists reported by Renovis and Hydra Biosciences and are still widely used as pharmacological tools in basic research [80,81]. A commonly used TRPV4 antagonist, RN-1734, was not used in our experiments because it inhibits TRPV4 with IC_50_ of 2.3, 3.2, and 5.9 µM in HEK293 expressing human, rat, or mouse TRPV4, respectively, while HC 067047 decreases TRPV4 activity at much lower concentrations with IC_50_ of 17, 48, and 133 nM, also showing much lower potency for inhibiting human TRPM8 with IC_50_ of 368 and 780 nM, respectively. HC-067047 also displays high selectivity with regard to TRPV4; its IC_50_ value is ~10-fold higher for TRPM8. Moreover, 1 μM HC-067047 caused complete inhibition of mouse TRPV4 [82]. Using only one TRPV4 antagonist is a clear limitation of this present study. Thus, confirming that a second blocker, such as RN-1734 or, even better, the most recently developed potent benzimidazole TRPV4 antagonist with the IC_50_ value of 22.65 nM [81], has similar effects that would be important in future studies.

It is noteworthy that in mesenteric arteries, and vessels of the systemic circulation, a novel mechanism for the regulation of blood pressure was suggested, whereby TRPV4 channels were involved and played roles dissimilar to PA [83]. In this case, it was shown that SMCs Gq protein signalling induces Ca^2+^ influx via endothelial TRPV4 channels for the purpose of negative feedback regulation of vasoconstriction. The authors explained the role of TRPV4 as a protective mechanism against over-vasoconstriction, where stimulation of Gq in SMCs leads to the synthesis of vasodilatory signalling factors in vascular endothelial cells. The α1-AR is highly expressed in the vascular system and plays a crucial role in the control of systemic blood pressure. It is well established that the α1-AR–TRPC3/TRPC6 complex is likewise important for the regulation of the contractile activity of blood vessels [84]. Interestingly, activation of α1-Ars in SMCs produces Ca^2+^ and IP_3_ as second messengers that, in turn, stimulate endothelial TRPV4 sparklets activity. An endothelial TRPV4/intermediate/small conductance Ca^2+^-sensitive K^+^ channels complex was supposed to act as the main negative feedback vasoconstriction regulator.

Determining a possible source of Ca^2+^ for the contraction phase is a more complicated task. Our previous results [12] have shown that the relaxation phase was independent of extracellular Ca^2+^. Thus, it persisted in both normal and Ca^2+^-free solutions, whereas the contraction phase disappeared in the absence of extracellular Ca^2+^. Surprisingly, we found a link between TRPV4 and Ca_L_ since the second phase of response to TRPV4 activation changed to vasorelaxation in case of Ca_L_ inhibition. Obviously, more than one agent is involved in the development of this complex reaction. First, these are adrenoceptors, the pre-activation of which is a necessary condition. Second, the other participants are Ca_L_, confirmed with the use of their selective blocker, nifedipine. Third, there is the apparent involvement of Ca^2+^ stores, as our tests using caffeine have shown (Figure 3).

TRPV4 channels are important regulators of vascular function and could be therapeutically targeted in vascular diseases [65]. Therefore, a more in-depth understanding of the mechanisms of their activation and interaction with other membrane protein complexes, which are currently only partially known, is an important step towards elucidating their role in various pathophysiological processes. Earley and his team demonstrated that Ca^2+^ influx through TRPV4 channels in myocytes activates BK_Ca_ channels and opposes pressure-induced constriction of cerebral arteries [5]. Moreover, Chen et al. [85] demonstrated that the α1AR–PKCα–TRPV4 and TRPV4–BK_Ca_ channel signalling microdomains are impaired in hypertension. Thus, they concluded that TRPV4 channel-mediated signalling could be targeted in order to reduce vasoconstriction and lower blood pressure in hypertension. TRPM8 channels are also highly expressed in various vascular beds, including rat aorta, mesenteric artery, femoral artery, and tail artery, where they can contribute to vascular tone regulation [14].

When TRPV4 or TRPM8 channels open Na^+^ and Ca^2+^ enter the cell, causing membrane depolarisation (Figure 4A and Figure 5A), which could then result in voltage-dependent activation of Ca_L_. Some generalisations can be made considering that activation of different types of TRP channels and in different blood vessels, pulmonary and systemic, produce highly similar membrane potential responses. The current paradigm states that Ca^2+^ sparks/STOCs coupling causes vasorelaxation. However, the novel “mixed” type of membrane potential responses reported here strongly implies that activation of BK_Ca_ channels secondary to TRP activation (STOCs shown in Figure 6 and Figure 7) can just as well, under certain conditions, promote Ca^2+^ entry via Ca_L_ channels and vasoconstriction. This hypothesis seems to be paradoxical, but let us consider, first of all, that BK_Ca_ activity that shows up as frequent spontaneous hyperpolarisations superimposed on slow TRP-induced depolarisation clearly limits the extent of membrane depolarisation. This is important since Ca^2+^ enters vascular myocytes not during action potential discharge, as in excitable cells, but via the mechanism known as “window” current. This current is maximal at the intersection of steady-state activation and inactivation curves of L-type Ca^2+^ channels. We found that in TA myocytes, this “window” Ca^2+^ current is maximal at −20 mV [13] so that excessive depolarisation (e.g., positive to −20 mV) would reduce rather than enhance Ca^2+^ entry via Ca_L_ channels. A second factor that deserves further quantitative evaluation is that frequent and fast hyperpolarisations when Ca_L_ channels are already activated are expected (i) to counteract voltage-dependent inactivation of L-type Ca^2+^ channels that inevitably develops at steady membrane depolarisation and/or (ii) to enhance Ca^2+^ entry simply due to frequent momentary increases in the electrochemical driving force for Ca^2+^. Thus, many interesting questions still remain, calling for further research in this direction.

## Data Availability

The datasets generated and analysed during this current study are available from the corresponding author upon reasonable request.
